# High prevalence of zero-dose children in underserved and special setting populations in Ethiopia using a generalize estimating equation and concentration index analysis

**DOI:** 10.1186/s12889-024-18077-w

**Published:** 2024-02-23

**Authors:** Gashaw Andargie Biks, Fisseha Shiferie, Dawit Abraham Tsegaye, Wondwossen Asefa, Legese Alemayehu, Tamiru Wondie, Meseret Zelalem, Yohannes Lakew, Kidist Belete, Samson Gebremedhin

**Affiliations:** 1 Project HOPE, Ethiopia Country Office, Addis Ababa, Ethiopia; 2 Project HOPE Headquarter, Washington, D.C USA; 3 School of Public Health, Addis Ababa University, Addis Ababa, Ethiopia; 4 Maternal and Child Health, Minister of Health, Addis Ababa, Ethiopia; 5 USAID, Addis Ababa, Ethiopia

**Keywords:** Zero-dose coverage, 12–35 months children, Hard-to-reach underserved settings, GEE generalized estimating equation, Pastoralist, Developing regions

## Abstract

**Background:**

Globally, according to the World Health Organization (WHO) 2023 report, more than 14.3 million children in low- and middle-income countries, primarily in Africa and South-East Asia, are not receiving any vaccinations. Ethiopia is one of the top ten countries contributing to the global number of zero-dose children.

**Objective:**

To estimate the prevalence of zero-dose children and associated factors in underserved populations of Ethiopia.

**Methods:**

A cross-sectional vaccine coverage survey was conducted in June 2022. The study participants were mothers of children aged 12–35 months. Data were collected using the CommCare application system and later analysed using Stata version 17. Vaccination coverage was estimated using a weighted analysis approach. A generalized estimating equation model was fitted to determine the predictors of zero-dose children. An adjusted odds ratio (AOR) with 95% confidence interval (CI) and a *p*-value of 0.05 or less was considered statistically significant.

**Results:**

The overall prevalence of zero-dose children in the study settings was 33.7% (95% CI: 34.9%, 75.7%). Developing and pastoralist regions, internally displaced peoples, newly formed regions, and conflict-affected areas had the highest prevalence of zero-dose children. Wealth index (poorest [AOR = 2.78; 95% CI: 1.70, 4.53], poorer [AOR = 1.96; 95% CI: 1.02, 3.77]), single marital status [AOR = 2.4; 95% CI: 1.7, 3.3], and maternal age (15–24 years) [AOR = 1.2; 95% CI: 1.1, 1.3] were identified as key determinant factors of zero-dose children in the study settings. Additional factors included fewer than four Antenatal care visits (ANC) [AOR = 1.3; 95% CI: 1.2, 1.4], not receiving Postnatal Care (PNC) services [AOR = 2.1; 95% CI: 1.5, 3.0], unavailability of health facilities within the village [AOR = 3.7; 95% CI: 2.6, 5.4], women-headed household [AOR = 1.3; 95% CI:1.02, 1.7], low gender empowerment [AOR = 1.6; 95% CI: 1.3, 2.1], and medium gender empowerment [AOR = 1.7; 95% CI: 1.2, 2.5].

**Conclusion:**

In the study settings, the prevalence of zero-dose children is very high. Poor economic status, disempowerment of women, being unmarried, young maternal age, and underutilizing antenatal or post-natal services are the important predictors. Therefore, it is recommended to target tailored integrated and context-specific service delivery approach. Moreover, extend immunization sessions opening hours during the evening/weekend in the city administrations to meet parents’ needs.

## Introduction

Childhood vaccination is a cornerstone in safeguarding the health and wellbeing of our future generations. However, there exists a population of children who have not received any routine vaccines, commonly known as zero-dose children. These vulnerable individuals lack the first dose of the diphtheria-tetanus-pertussis containing vaccine (DTP1/penta1). The under-immunized child, on the other hand, refers to those who have not received the crucial third dose of diphtheria, tetanus, and pertussis (DTP) [1, 2].

According to the 2023 World Health Organization (WHO) immunization report, a staggering 14.3 million children fall into the category of zero-dose children [3]. Majority of these children reside in low- and middle-income countries, with particularly high numbers found in African and South-East Asian regions. Ethiopia, unfortunately, is one of the top contributors to this alarming statistic [3]. The country exhibits significant geographic disparities in vaccine coverage, as evidenced by studies highlighting the lowest proportion of immunized children in regions like Somali and Afar. Conversely, the Amhara region boasts the highest proportion of immunized children [4, 5].

Interestingly, communities with predominantly agrarian lifestyles seem to demonstrate higher immunization coverage. This phenomenon can be attributed to factors such as increased access to vaccination services, improved infrastructure, enhanced socio-economic status, high literacy rates, and better information dissemination [6, 7]. Furthermore, women in these regions may play a pivotal role in decision-making processes regarding immunization. In addition, those who possess a medium or higher wealth status, reside closer to healthcare facilities, exhibit comprehensive knowledge about pregnancy complications, and hold positive perceptions towards immunization tend to achieve higher rates of coverage [8, 9]. On the contrary, regions with lower immunization coverage predominantly consist of pastoralist and semi-pastoral areas, as supported by previous Ethiopian Demographic and Health Surveys [4, 5]. These regions face unique challenges, including limited access to healthcare services, resulting in higher mortality rates among under-five children, particularly those who are under-immunized or devoid of vaccinations altogether [10, 11]. The lack of robust infrastructure, limited distribution networks, and poor-quality services might compound the difficulties faced by these regions [12].

In addition to the factors mentioned above, various literatures have been identified as contributing to the zero-dose status among children. These include the sex of the child [13, 14], the age of the mother [15–17], access to antenatal (ANC) and postnatal care (PNC) follow-up services, and proximity to vaccination sites [18]. However, it should be noted that the association between sex and zero-dose status is occasional, as most countries exhibit minimal differences in childhood immunization rates based on gender [19–22]. Additional factors include ethnicity [23] and religion [24]. Other researches also indicates that the place of residence and distance from health institutions serve as predictors of child immunization rates [25–27]. Wealth status also influences vaccination coverage, with lower coverage observed among children from households with lower wealth status in Ethiopia, Nigeria, and Pakistan [28–31]. Conversely, in Brazil, lower vaccination rates are observed among children from households with higher wealth status [32]. The existence of these disparities across countries and localities highlights the need for a deeper understanding of context-specific factors and inequalities in childhood vaccination coverage [33].

The global community, through the Immunization Agenda 2030, aims to eradicate this inequity by prioritizing the immunization of zero-dose children in the next five years. The goal is to reduce the number of zero-dose children by 25% by 2025 and by 50% by 2030, aligning with the closing year of the sustainable development goals (SDGs) [34]. Achieving the objectives outlined in the WHO/UNICEF immunization agenda (IA2030) [35, 36] and the SDGs necessitates comprehensive research and focused attention on zero-dose children.

There is a surge in global attention towards the Global Alliance for Vaccines and Immunisation (Gavi 5.0) “Leaving no one behind” Strategy, which aims to reduce the number of zero-dose children by 50% by 2030. This strategy is committed to providing equitable and sustainable vaccination services for all, including underserved urban areas, hard-to-reach communities, and conflict-affected populations [37–39]. The Ethiopian Health Sector Transformation Plan (HSTP) II (2021-25) also places a strong emphasis on controlling vaccine-preventable diseases (VPDs) and aims to increase full vaccination coverage from 44 to 75% [40]. In line with these goals, the National Expanded Program on Immunization (EPI) Comprehensive Multi-year Plan (2021–2025) sets notable objectives, including achieving 90% full vaccination coverage at the national level and 85% coverage in every district [41]. Currently, there is no documented evidence regarding factors contributing to vaccination coverage differences within different regional contexts in Ethiopia.

Therefore, this study was conducted to estimate the proportion of zero-dose children who live in remote, conflict-affected, and underserved settings, and to identify the associated factors in Ethiopia. Thus, it will be important to design robust programs to sustainably reach zero-dose children to reach 2030 targets while avoiding a “one size fits all” approach in these underserved and special setting populations of Ethiopia.

## Methods

### Study design and settings

A cross-sectional vaccination coverage survey was conducted in an underserved setting of Ethiopia in June 2022. The samples were taken from the following study areas: pastoralist regions (Afar, Somali, Gambella, Oromia, Southwest, and Southern Nations, Nationalities, and People’s Region [4] regions), developing regions (Afar, Somali, Gambella, and Benishangul Gumuz (BG)), newly-established regions (Sidama and Southwest), hard-to-reach areas in agrarian regions (Amhara, Oromia, and SNNP regions), conflict-affected areas (Amhara, Afar, Oromia, and BG), urban slums in major towns and cities (Addis Ababa, Dire Dawa, Harar, Bahir Dar, Hawassa, and Adama), and special populations (refugees and internally displaced peoples (IDPs).

### Study participants

The target population were under-five children who reside in underserved, remote, or conflict-affected areas of Ethiopia. The study population consisted of mothers/caregivers and children between the ages of 12–35 months in the study settings.

### Sample size determination

The total sample size for each target population was calculated using Cochran’s Single Population Proportion Sample Size Formula assuming 95% confidence level, 4% margin of error, 16% prevalence of zero-dose children [4, 5], and 10% compensation for possible non-response. Accordingly, a sample size of 360 was required for each of the population domains. Based on our analysis of Ethiopian DHS 2016 and Mini DHS 2019 data, an average of 12 children aged 12–35 months are available per enumeration area. Therefore, a minimum of 30 enumeration areas (EAs) were required to recruit 360 mothers who had children aged 12–35 months for each target population, assuming all children in the EA would be eligible for inclusion in the study. Initially, we planned to include 4,080 children from the 340 EAs (a minimum of 360 children per target population domain) in the survey. However, 3,646 mothers with eligible children aged 12–35 months from the 304 EAs were included due to active conflict mainly in the northern part of the country. Nevertheless, the total sample size was large enough to allow subgroup analysis based on sex and age groups.

### Sampling procedure

The sample survey was representative of the selected target populations. Study subjects were selected through a stratified sampling method in a two-step procedure according to the recommendations by the WHO for conducting vaccination coverage cluster surveys [42]. This assured the comparability of the survey data with large scale standardized surveys such as the DHS and Multiple Indicator Cluster Surveys. First, 304 EAs were randomly selected from the total EAs available for each target population domain. The EAs delineated by the Central Statistical Agency of Ethiopia for the recent census were used as a sampling frame. In the case of urban slums, hotspots in Addis Ababa, Adama, Bahir Dar, Hawassa, Harar, and Dire Dawa towns were located, delineated, and EA maps were drawn by experienced cartographers. In the case of IDPs and refugee camps, villages or clusters were considered as EAs. Next, all of the eligible children in each EA were listed. Then, 12 children aged 12–35 months were ultimately selected using a smartphone-based random number generator through a stratified sampling approach.

### Data collection procedures and quality assurance

Mothers/caregivers were interviewed using a pre-tested questionnaire prepared in five local languages which included Amharic, Afan Oromo, Somali, Afar, and Sidama. Survey data were collected by 48 experienced enumerators and 24 supervisors using the CommCare digital app [43], an open-source and user-friendly application system that is interoperable with major data analytics and visualization software. The CommCare app system helped us to collect individual child-level and household information, to ensure high-quality data collection, cleaning, and monitoring in real time.

Data collectors were recruited based on educational status (at least diploma holder in a health-related discipline), previous work experience in national surveys, and familiarity with the CommCare digital App. The survey team received five days of intensive training on the sampling approach, proper interviewing techniques, and field practice in reviewing child immunization cards and the use of CommCare digital app for individual child-level immunization data gathering. Each data collector was allowed to collect data from no more than six children per day. To validate the quality of the data, one-third (33%) of all the study participants were re-interviewed by the supervisors. The research team throughout the survey implementation period also closely monitored the data entered to CommCare.

### Variables and operational definitions

Zero-dose children were defined as those children lacking DTP1/ penta1 in this analysis. The proportions of zero-dose children was calculated as those missing DPT1 containing vaccine /penta1 [1, 2].

Underserved and special setting populations receive fewer healthcare services, including immunization services; face economic constraints to bring their children to vaccination services areas; face cultural and/or linguistic barriers to accessing immunization healthcare services; lack familiarity with the healthcare delivery system and; live in locations where providers aren’t readily available or physically accessible, such as pastoralist regions, developing regions (Afar, Somali, Gambella, and BG), newly-established regions of Sidama and South West Ethiopia Peoples’ Region regions, conflict-affected areas, underserved urban population including slum area, hard-to-reach areas in agrarian regions (Amhara, Oromia, and SNNP regions), IDPs, and refugees.

Immunization status was determined by self-reports from mothers/caregivers, immunization card reviews, and facility-based record reviews, as recommended by the WHO [44]. In areas where the mother or caregiver presented an immunization card, the child’s immunization status was based on vaccination card review. Where an immunization card was not available, the immunization status was assessed according to mothers’/caregivers’ self-reports/recalls. Previous studies have proven this method to be a reliable assessment of zero-dose coverage in resource-limited settings with poor documentation of childhood immunization [44].

### Women’s empowerment

The quest for gender equality and women’s empowerment has led us to devise methods of measuring women’s influence in domestic decision making. In this study, a composite scale was developed to capture the extent of women’s power in various areas of their lives. By examining women’s reported authority in six specific domains, including major household purchases, expenditure of their own income, expenditure of their partner’s income, visits to families or relatives, and decisions regarding healthcare for themselves and their children, a comprehensive and nuanced picture of women’s empowerment emerged.

To assign scores on the composite scale, each component was carefully assessed. The coding system employed a binary approach, with a value of “0” assigned when decisions were made solely by the woman’s partner, and a value of “1” assigned when decisions were made either by the woman herself or jointly with her partner. Utilizing this coding system, a maximum score of 6 could be obtained, indicating a high level of women’s empowerment.

Ultimately, the scores were categorized into three ordinal categories: low, medium, and high. For individuals whose scores ranged from 0 to 2, they fell within the low empowerment category. Those with scores of 3 or 4 were classified as having medium empowerment, while individuals with scores of 5 or 6 were deemed to have high empowerment. This categorization allowed for a comprehensive understanding of the level of empowerment experienced by women in the context of domestic decision making.

The use of a composite scale to measure women’s empowerment offers a multidimensional perspective on the distribution of power within households. By examining different aspects of decision making, researchers were able to capture the complexities of women’s agency and autonomy in shaping their lives. The scale provided a systematic and comprehensive approach to understanding the extent of women’s influence, offering insights into areas where gender disparities may persist, as well as areas where progress has been made.

Wealth index was categorized based on ownership of valuable assets and livestock, size of land for agriculture and housing purposes, materials used for house construction, and access to basic social services such as electricity, banking, improved water sources, and bodily waste disposal methods. A total of 41 variables were reduced into nine factors using principal component analysis [45]. The components were further summated into a score and ultimately classified into five quintiles (poorest, poorer, middle, richer, and richest).

### Data management and statistical analysis

Data were exported from CommCare to Microsoft Excel [43] for cleaning. Afterwards, data were transferred to Stata 17 [46] for advanced statistical analyses.

The primary outcome of interest was the proportion of children who had not received any vaccines by 12 months of age. Predictor variables of zero-dose children included wealth index, living in urban slums, younger child age, child sex, mothers/caregivers employment status, mothers/caregivers educational status, mothers/caregivers marital status, skilled birth attendance, number of ANC visits, use of PNC services for the index child, age of mothers, decision categorized, sex of household heads, availability of public health facilities within the kebele, geographical disparities such as hard-to-reach and remote areas, urban slums, IDPs, pastoralist regions, developing and newly-formed regions, refugees camps, and conflict-affected.

We used frequency tables and calculated percentages to describe the data and ran χ2 to examine the relationship between each of the predictor variables with the primary outcome variable. We used t-test to assess statistical differences in the mean scores for knowledge of routine immunization. We dichotomised aggregate scores on awareness of routine immunization into satisfactory knowledge (10 points and above) and poor knowledge (less than 10 points) prior to inclusion in the regression model. A bivariate analysis was carried out to examine the relationship between zero dose and the potential predictors without adjusting for other covariates. Then all potential predictors with a *p*-value less than 0.20 in the univariate analysis were used to include more potential predictors. In addition, factors previously reported to be associated with zero dose were entered into a generalized estimating equation (GEE) regression model to examine their effects simultaneously. In the GEE analysis model, possible associated factors were examined for evidence of collinearity which was reflected either by the changes in the direction of effect between the univariate and multivariate analysis or implausible standard errors for a particular variable. A GEE model was fitted to estimate the adjusted odds ratio (AOR) with 95% confidence interval (CI) while adjusting for mother’s age, marital status, mothers’ educational status, mothers’ occupation, wealth index, and sex of the index child. Data were analysed using Stata 17 for Windows.

Since the study subjects were selected through a stratified sampling method in a two-step procedure, the vaccination coverages were estimated using a weighted analysis approach to balance weighted and unweighted sample size, linearization of post-stratification. The study divided the units in a target population such as urban and rural then hard-to-reach/agrarian, developing and pastoralist, newly formed regions, IDPs, and refugee camps strata that were homogeneous within and heterogeneous between in terms of the survey measures of interest. A fraction of the overall sample was allocated to each of the strata, often to minimize the variance of estimates. Select samples within each of the strata was simple random samples to ensure representation from all strata. In general, we combined the estimates from the strata into overall estimates to compensate for the over- or under-sampling of specific cases or for disproportionate stratification. Finally, linearization of post-stratification weighted analysis was used to allow for different probabilities of selection or various levels of non-response in our study [47].

The concentration curve is obtained by plotting the cumulative proportion of outcome variables (vaccination status) on the y-axis against the increasing percentage of the population ranked by the socioeconomic indicator (wealth index) on the x-axis. The curves show that whether the socio-economic status related inequality in the outcome variable (on x-axis) prevails or not [48, 49]. If the curve is above the line of equality that means the index value is negative; hence it shows that the outcome variable is disproportionally concentrated among the poor and vice-versa [48, 49]. The concentration index is defined as twice the area between the concentration curve and the line of equality. The concentration index measures the inequality of one variable (zero-dose prevalence) over the distribution of another variable (wealth index) [50]. The index ranges from − 1 to + 1, where the index value of 0 (zero) shows no socioeconomic inequality [50]. However, the positive value of the index shows pro-rich inequality and vice-versa. Additional on either scale higher the value, the higher the extent of socioeconomic inequality. Concentration index, Wagstaff’s index, and Erreygers index are all binary variables that condition the level of absolute inequality on the most unequal society, although their definitions of that state differ [51].

### Ethical considerations

This survey was implemented in compliance with national and international ethical guidelines. The protocol was ethically cleared by the institutional review board of the Ethiopian Public Health Institute. Administrative clearances were secured from each regional health bureau and data were collected after taking informed consent from the study participants. To prevent the risk of COVID-19 transmission, precautionary measures including use of hand sanitizers, physical distancing, and ventilation of interview settings were practiced. To maximize beneficence, all under-immunized children were referred to the nearest health facility.

## Results

### Sociodemographic characteristics of study participants

#### Mothers/Caregivers

A total of 3,646 mothers/caregivers were interviewed with a response rate of 97.7%. Close to three-fourths 2,657 (72.9%) of the respondents were drawn from rural areas. The mean (± standard deviation) age of the respondents was 28.7 (± 6.7) years and 2,007 (55.05%) were between 25 and 34 years of age. Little more than half of the respondents had no formal education 1,892 (51.89%) and 2,551 (69.97%) were unemployed or not working at the time of survey. The vast majority 3,346 (91.8%) of the mothers/caregivers were married/co-habiting, and about 41.36% of their partners had no formal education. About one-in-ten of the households were female-headed, 39% of mothers/caregivers had four or more ANC visits, 61.89% had skilled birth attendants, and 49.63% had received PNC services for the index child.

#### Children

Regarding the profile of participating children, boys (51.45%) were slightly more represented than girls (48.55%) and nearly half (49.26%) of participants were between 12 and 23 months of age. The remaining (50.74%) were 24–35 months of age (Table 1).

**Table 1:** Socio-demographic characteristics of zero-dose children in underserved and special setting population in Ethiopia, June 2022

Characteristics	Frequency	Percent (%)
Region*		
Afar	588	16.13
Amhara	312	8.56
Oromia	372	10.20
Somali	336	9.22
Benishangul Gumuz	216	5.92
SNNPR	300	8.23
Sidama	179	4.91
Southwest Ethiopia	181	4.96
Gambella	360	9.87
Harari	60	1.65
Addis Ababa	192	5.27
Dire Dawa	60	1.65
Amhara urban	60	1.65
Oromia urban	59	1.62
Sidama urban	60	1.65
Refugees	311	8.53
**Wealth Index**		
Richest	729	19.99
Richer	731	20.05
Middle	728	19.97
Poorer	729	19.99
Poorest	729	19.99
**Child’s sex**		
Boy	1,876	51.45
Girl	1,770	48.55
**Child’s age (in months)**		
12–23	1,796	49.26
24–35	1,850	50.74
Respondents’ age* (in years)		
15–24	857	23.51
25–34	2,007	55.05
35–44	596	16.35
45 or above	91	2.50
DK	95	2.61
**Respondent’s educational status**		
No formal education or preschool	1,892	51.89
Primary education	718	19.69
Secondary education	885	24.27
Tertiary education	151	4.14
**Paternal educational status**		
No formal education or preschool	1,508	41.36
Primary education	555	15.22
Secondary education	878	24.08
Tertiary education	379	10.39
No husband	300	8.23
DK	26	0.71
**Marital status**		
Married/Living together	3,346	91.77
Divorced	102	2.80
Separated	78	2.14
Widowed	67	1.84
Not ever married	53	1.45
**Household head**		
Male-headed	3,310	90.78
Female-headed	336	9.22
**Place of residence**		
Rural	2,657	72.87
Urban	989	27.13
**Mother’s/Caregiver’s employment status**		
Not working	2,551	69.97
Working	1,095	30.03
**Number of Under five children**		
One child	1,632	44.76
Two children	1,519	41.66
Three and above	495	13.58
**Household size**		
Less than six	1,914	52.50
6 or above	1,732	47.50
**Mother who had ≥ 4 Ante-natal Care (ANC) Visits**		
Yes	1,318	38.57
No	2,099	61.4
**Mother who had received Post-natal Care (PNC)**		
Yes	1,697	49.63
No	1,722	50.37
**Skilled Birth Attendance (SBA)**		
Yes	2,116	61.89
No	1,303	38.11
**Availability of health facility in the kebele**		
Yes	3,383	92.79
No	263	7.21
**One-way walking distance to the nearest health facility**		
30 min or less	2,044	56.06
30 to 60 min	817	22.41
More than an hour	785	21.53
**Gender empowerment**		
Low	436	11.96
Medium	546	14.98
High	2,364	64.84
Not Married	300	8.23

*Respondents were mothers or caregivers

### Ownership of vaccination card

The possession of a vaccination card can serve as a valuable proxy indicator for assessing an individual’s access to vaccination services. Within our study sample, it was found that 60.8% of children aged 12–35 months possessed a vaccination card, indicating their utilization of vaccination services. However, notable variations were observed across regions, with ownership rates being significantly lower in developing regions (37.8%) and pastoralist settings (50.5%), particularly in the Somali region (26.5%). On the other hand, a significantly higher proportion of children from urban settings (97.1%) were found to own a vaccination card (Table 2).

**Table 2:** Vaccination card ownership among children 12–35 months in remote, underserved, and special settings in Ethiopia, June 2022

Settings	% Ownership of vaccination card
**Developing regions (n = 1,380)**	37.8
Afar (n = 516)	62.4
Benishangul Gumuz (n = 168)	41.7
Gambella (n = 360)	79.7
Somali (n = 336)	26.5
**Newly formed regions (n = 418)**	**60.5**
Sidama (n = 179)	44.7
Southwest (n = 181)	67.1
**Hard to reach areas in major regions (n = 786)**	**68.2**
Amhara (n = 240)	81.7
Oromia (n = 300)	67
SNNP (n = 300)	61.3
Conflict affected settings1 (n = 276)	68.1
Pastoralist populations2 (n = 1,344)	50.5
Underserved settings in predominately urban regions and city administrations (n = 491)	97.1
Urban slums (n = 443)	97
Special populations	
IDPs (n = 264)	68.2
Refugees (n = 311)	67.5
Total (n = 3,646)	60.8

### Prevalence of zero-dose and under-immunized children

**Fig. 1:**
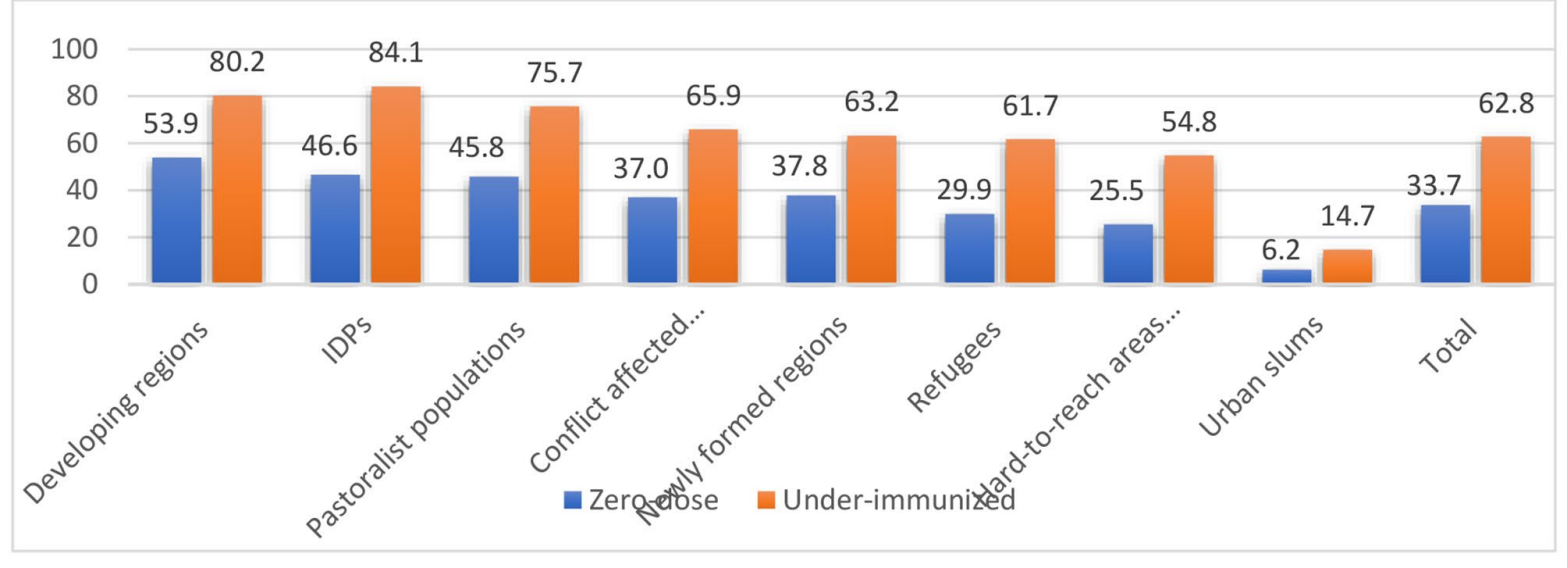
Prevalence of zero-dose and under-immunized children in remote, underserved, and special settings in Ethiopia, June 2022

The estimated pooled prevalence of zero-dose and under-immunized children was 33.7% (95% CI: 24%, 65%) and 62.8% (95% CI: 55%, 69.5%) respectively (Fig. 1). Developing regions including Afar, Somali, Gambella, and BG had the highest (53.9%) prevalence of zero-dose children. The lowest prevalence of zero-dose (6.2%) and under-immunized children (14.7%) were found in urban slums. Among the eight population domains studied, Penta-1 coverage rates were higher (93.8%) in urban slums. Conversely, Penta-1 rates in developing and newly formed regions were 46.1% and 62.2%, respectively (Fig. 1).

### Predictors of zero-dose prevalence

Based on the bivariate analysis poor/poorest wealth index, living in urban slums, younger child age, child sex, mothers/caregivers employment status, mothers/caregivers educational status, mothers/respondents marital status, skilled birth attendance, number of ANC visits, use of PNC services for the index child, age of mothers/caregivers, decision categorized, sex of household heads, availability of public health facilities within the kebele, geographical disparities such as hard-to-reach and remote areas, urban slums, IDPs, pastoralist regions, developing and newly-formed regions, refugees camps, and conflict-affected areas were statistically significant predictors of zero-dose status (Table 3).

**Table 3:** Factors associated with Zero dose coverage in underserved settings in Ethiopia by socioeconomic, maternal, and child characteristics in Ethiopia, June 2022

Socio-economic status (*n* = 3646)	Took Penta1 Freq. (%)	Zero dose Freq. (%)	COR	AOR
**Wealth status**				
Richest	405(78.92)	108(21.08)	1	1
Richer	565(76.23)	176(23.77)	1.18 (0.99-1.42)	1.32(0.81–2.14)
Middle	524 (69.96)	225 (30.0)	1.57(1.19–2.09)	1.66(0.91–3.03)
Poorer	520 (60.84)	335 (39.16)	2.34(1.79–3.06)	1.96(1.02–3.77)
Poorest	403(51.23)	384 (48.77)	2.95(2.26–3.87)	2.78(1.70–4.53)
**Hard to reach areas**				
Yes	602(76.59)	184(23.41)	1.74 (1.05–2.87)	1.64 (0.88–3.06)
No	1,862 (65.10)	998 (34.90)	1	1
**Pastoralist Regions**				
Yes	776(57.74)	568(42.26)	2.23(1.06–4.68)	0.66(0.34–1.27)
No	1,688(73.33)	614(26.67)	1	1
**Developing regions**				
Yes	807(58.48)	573(41.52)	2.72 (1.36–5.44)	4.49(1.44–14.02)
No	1,657(73.12)	609(26.8)	1	1
**Refugees**				
Yes	218(70.10)	93(29.90)	0.83(0.56–1.23)	2.38(0.96–5.90)
No	2,246(67.35)	1,089(32.65)		1
**Conflict affected areas**				
Yes	174 (63.04)	102 (36.96)	1.01 (1.00-1.07)	1.00(0.63–1.43)
No	2,290 (67.95)	1,080 (32.05)	1	1
**Urban slum areas**				
Yes	24 (92.55)	2(7.45)	1	1
No	2,394 (66.13)	1,226 (33.87)	6.36(2.30–17.60)	1.19 (0.43–3.35)
**Maternal educational status**				
No formal education	1,372 (63.58)	786 (36.42)	3.24(0.74–14.19)	1.17(0.38–3.62)
Primary education	532(67.49)	256(32.51)	3.02(0.65–14.15)	1.33(0.38–4.74)
Secondary education	441(71.56)	175 (28.44)	2.65(0.68–10.38)	1.73(0.43-7.00)
Tertiary education	73(87.04)	11(12.96)	1	1
**Maternal employment**				
Working	1,019(65.85)	529(34.15)	1.31(0.96–1.80)	1.43(1.00-2.05)
Not working	1,399(66.65)	700(33.35)	1	1
**Paternal educational status**				
No formal education	1,057(63.14)	617(36.86)	1.40 (1.13–1.75)	1.26(1.00-1.59)
Primary education	471(65.78)	245(34.22)	1.42(1.07–1.89)	1.27(0.93–1.75)
Secondary education	511(73.32)	186(26.68)	1	1
Tertiary education	175(81.29)	40(18.71)	0.61(0.28–1.33)	0.71 (0.39–1.26)
No Husband	195(58.45)	139(41.55)	-	-
DK	9(85.46)	2(14.54)	-	-
**Place of residence**				
Urban	504(74.41)	173(25.59)	1	1
Rural	1,914 (64.46)	1,055 (35.5)	1.46(1.11–1.92)	1.09(0.80–1.49)
**Child Age**				
12–23	1,241(67.14)	607(32.8)	1	1
24–35	1,177 (65.46)	621(34.54)	1.06 (0.95–1.18)	1.04(0.92–1.18)
**Child sex**				
Boys	1,324 (66.73)	660 (33.27)	1	-
Girls	1,094 (65.82)	568 (34.18)	1.02 (0.89–1.17)	-
**Number of under-five children**				
One	1,195 (68.63)	546 (31.37)	1	1
Two	1,072(66.81)	533(33.19)	1.00(0.88–1.15)	0.79(0.39–1.60)
Three or more	151 (50.26)	150(49.74)	1.53(1.11–2.11)	1.11(0.26–4.85)
**Marital status**				
Currently not married	195(58.45)	139(41.55)	1.51(1.34–1.69)	2.39(1.72–3.33)
Married/ living together	2,223(67.10)	1,090(32.90)	1	1
**Maternal age**				
15–24	570(65.10)	305(34.90)	1.20(1.08–1.34)	1.19(1.08–1.31)
25–34	1,346 (8.34)	623 (31.66)	1	1
35–44	368 (64.36)	204 (35.64)	1.10(0.68–1.63)	1.20(0.65–2.24)
45 or above	57(53.78)	48(46.22)	1.62(1.17–2.25)	1.17(0.47–2.91)
DK	79(62.30)	48(37.70)		-
**ANC visits**				
4 or more	1,009(77.36)	295(22.64)	1	1
Less than 4 visits	1,276 (60.38)	837 (39.62)	1.73 (1.58–1.91)	1.29(1.19–1.41)
**SBA**				
No	847(55.82)	670(44.18)	2.00(1.39–2.90)	1.32(0.84–2.07)
Yes	1,439(75.67)	463(24.33)	1	1
**PNC**				
No	1,135(58.35)	810(41.65)	2.20(1.67–2.91)	2.09(1.45–3.02)
Yes	1,151(78.10)	323(21.90)	1	1
**Availability of health facility in the kebele**				
Yes	2,309(69.82)	998(30.18)	1	1
No	109(32.10)	231(67.90)	4.40(3.06–6.32)	3.70 (2.55–5.36)
**One-way walking distance to the nearest health facility**				
30 min or less	1,201(70.34)	507(29.66)	1	1
30–60 min	488(65.77)	254(34.23)	1.20(0.78–1.83)	(0.75–2.24)
More than an hour	728(60.89)	468(39.11)	1.52(1.14–2.03)	1.00(0.73–1.26)
**Gender empowerment**				
Low	262(58.76)	184(41.24)	1.64(1.28–2.12)	1.63(1.26–2.096)
Medium	271(58.95)	189(41.05)	1.62(1.24–2.11)	1.71(1.16–2.52)
High	1,691(70.20)	718(29.80)	1	1
No married	195(58.45)	139(41.55)	-	-

In the Multivariate generalized estimating equation analysis, predictors of zero-dose prevalence were identified. Children from the poorest households with wealth index inequalities being poorest compared to richest [AOR = 2.78; 95% CI: 1.70,4.53] and being poorer compared to richest [AOR = 1.96; 95% CI: 1.02, 3.77] were more likely to be zero-dose children. Additionally, mothers/caregivers who were employed/working in governmental institutions or other businesses activities were more likely to have zero-dose children [AOR = 1.43; 95% CI: 1.00, 2.05] compared to mothers who were unemployed. Mothers/caregivers who were not currently married/single were more likely to have zero-dose children [AOR = 2.39; 95% CI: 1.72, 3.33] compared to currently married mothers/caregivers. The odds of having zero-dose children were also found to be higher among mothers between the ages of 15–24 years, compared to older mothers [AOR = 1.19; 95% CI: 1.08, 1.31].

Mothers/caregivers who received less than four ANC visits were more likely to have zero-dose children [AOR = 1.32; 95% CI: 1.19, 1.41] compared to mothers who had less than four or more ANC visits. Similarly, mothers/caregivers who were not receiving PNC services were more likely to have zero-dose children [AOR = 2.09; 95% CI: 1.45, 3.02] compared to those who had received PNC services. The likelihood of having zero-dose children was higher among those who reported that health facilities were unavailable within their Kebele [AOR = 3.70; 95% CI: 2.55, 5.36] compared to their counterparts, the odds of having zero-dose children were higher for those with a male head of the household [AOR = 1.33; 95% CI: 1.02, 1.74] and for those from developing regions [AOR = 6.35 95%; CI: 2.57, 15.68] and newly established regions [AOR = 4.49; 95% CI: 1.44, 14.02]. 63% of children born to less-gender empowered women, those with restricted decision-making power, were found to be zero-dose children. Furthermore, 71% of children within households where women held medium levels of gender empowerment also fell into the same category (Table 2).

Table 4 presents wealth index inequality for children who were zero-dose among regions in underserved setting of Ethiopia during June 2022. The results indicated that wealth index inequalities were highly observed in Somali, Addis Ababa City Administration, BG, Dire Dawa, Southwest, and SNNP regions of the country.

**Table 4:** Show wealth index inequality among regions of Ethiopia, June 2022

Regions	Mean	Median	N (Population)
Afar	− 0.1659306	− 0.1094294	636
Amhara	− 0.1492822	− 0.1094294	372
Oromia	− 0.1758743	− 0.1094294	431
Somali	− 0.7481544	− 0.9103071	480
Benishangul Gumuz	− 0.3062662	− 0.5647229	216
SNNP	− 0.2127097	− 0.5647229	300
Sidama	0.0770098	− 0.1116236	239
SW	− 0.2220966	− 0.1116236	181
Gambella	0.0553038	− 0.1094294	479
Harari	− 0.4531486	− 0.5647229	60
Addis Ababa	− 0.6603757	− 0.9103071	192
Dire Dawa	− 0.2347906	− 0.5647229	60

### Concentration index and curve

**Fig. 2:**
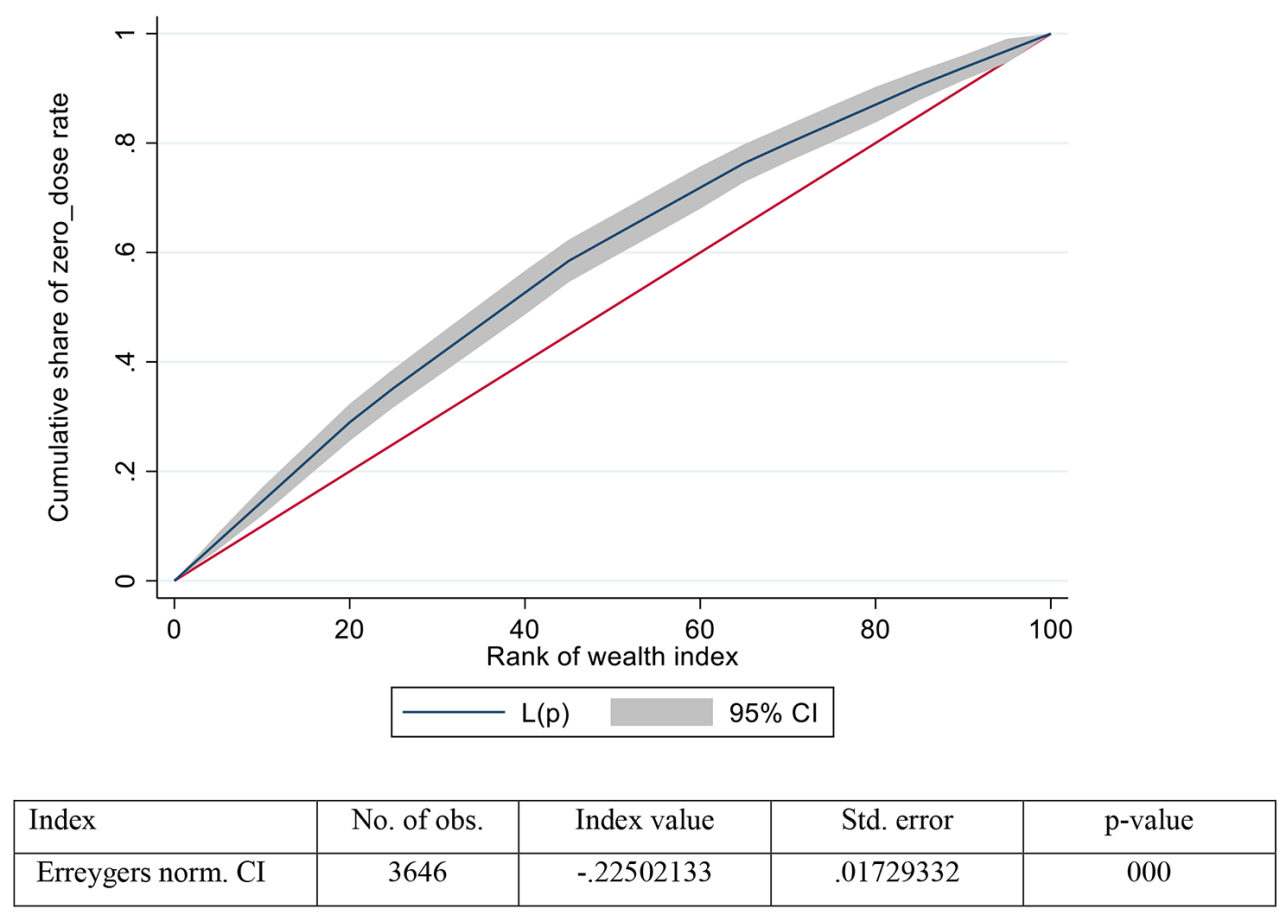
Concentration curve for zero dose children coverage against wealth index rank, in underserved and special setting population in Ethiopia (Project HOPE, zero dose project, 2022)

In our study, the concentration curve lies above the line of equality. This implies that zero-dose vaccination coverage is concentrated towards the lower-income proportion of the population in Ethiopia. This implies that the result characterised by a pro-rich distribution with respect to household wealth index with relatively higher vaccination coverage was observed among children from richer and richest households. In our context concentration index were negative and we can understand that zero dose is highly concentrated and more problematic among the poorest and poorer communities in the study settings (Fig. 2).

## Discussion

To our knowledge, this is the first national survey estimating the prevalence of zero-dose vaccination and examining the predictors among children aged 12–35 months in underserved settings of Ethiopia. The overall estimated prevalence of zero-dose children in the study settings was 33.7%. The general findings of the study were not similar to the WHO/UNICEF Estimates of National Immunization Coverage 2022 report which estimated that 30% of surviving infants in Ethiopia were zero-dose children [35]. However, it is pivotal to note that our findings do not align with the estimates provided by the esteemed World Health Organization (WHO) and the United Nations Children’s Fund 2022 report on national immunization coverage. Nonetheless, these two sets of data are incomparable due to several distinctive factors.

Firstly, the WHO/UNICEF estimates offer a comprehensive overview of immunization coverage at the national level, encompassing various regions and settings across Ethiopia. Our study, however, specifically hones on the underserved areas, shedding light on a specific demographic that requires immediate attention and tailored interventions. Moreover, the discrepancy arises from the differing age groups examined. While the WHO/UNICEF estimates focus on infants up to the age of 12 months, our study expands the scope to encompass children up to 35 months. By extending the age range, we capture a wider spectrum of children, providing a more holistic understanding of zero-dose vaccine prevalence in this vulnerable population.

Additionally, it is worth noting the study conducted by Nour et al., which utilized a systematic review and meta-analysis to estimate the overall prevalence of immunization coverage in Ethiopia. Their findings revealed a higher percentage of approximately 47.0% [3, 4]. While our study does not aim to directly contradict their results, it is essential to consider the differences in methodologies and specific populations studied. On the contrary, the prevalence of zero-dose children was greater than the findings in the studies by EHDS 2016 [4] and Mini EHDS 2019 (19%) Reports in Ethiopia [5]. Our estimates were also higher compared with a similar study done in sub-Saharan Africa countries (16.5%) [52] and another study done in the 92 low- and middle-income countries which found 7.7% of children had not received any vaccines [53]. In addition, a study done by Bosch-Capblanch et al., reported a prevalence of 9.9% zero-dose children aged 12–59 months in low- and middle-income countries [53]. The possible reason for this significant variation among the different population domains could be weak health infrastructure, distance to health facilities, availability of vaccine logistics, storage in the health institutions, especially at the health center and health post levels, and mobility patterns of the population making it difficult to deliver routine immunization services to the targeted children [54].

In our study, according to the sub-group analysis with different population domain, there were geographic variations in the magnitude of zero-dose and under-immunized children. Among the eight population domains studied, Penta-1 coverage rates (93.8%) were higher in urban slums. This might be due to increased access to vaccinations in urban versus rural areas [55]. Conversely, it was found that developing and newly formed-regions had the highest prevalence of zero-dose children of 53.9% and 37.8%, respectively. We understood that coverage varies widely among and within regions and different settings of Ethiopia. The findings of this study were also consistent with previous study results [56–58]. These findings highlight the variability of zero-dose coverage, both geographically and in terms of the characteristics of those populations, demonstrating the need for both regional and context-specific strategies for immunization services [34, 35]. This immunization coverage variation might be due to the differences in vaccination service access. For example, the odds of having a zero-dose child was higher among those who reported health facilities as unavailable within their village [AOR = 3.70; 95% CI: 2.55, 5.36]. Our study results were generally consistent with a previous study conducted in low and middle-income countries [57], for example Mozambique [59, 60], Zimbabwe [61], in Ethiopia Debre-Markos town [62], and Minjar-Shenkora district [63]. This finding indirectly implies that the health extension program and health development army have become ineffective in increasing access and awareness about child immunization and in reaching zero-dose children by tracing unreached and missed communities in the study settings [64, 65].

A multivariate analysis with GEE revealed that wealth index, mother’s/caregiver’s age, level of gender empowerment, mother’s/caregiver’s employment status, marital status, head of household sex, number of ANC visits, use of PNC services, availability of health facilities within the village, and type of region were found to be key factors affecting the prevalence of zero-dose children in underserved setting populations of Ethiopia.

Our study found that wealth index was a significant predictor of zero-dose coverage among children aged 12–35 months in the study settings. In this regard, we found that parents from rich and richest households were less likely to have zero-dose children than their low-income counterparts. Households who were in the richer and richest wealth quintiles are more likely to have access to good healthcare facilities [66]. Notably, the wealth index groups may have better economic and social influence and children from richer households appear to have higher odds of receiving early vaccination than children from poorer and poorest households [30]. A similar study found that socioeconomic and maternal/caregivers’ regional- and context-specific wealth index inequalities also pose a huge threat to early childhood vaccination [59, 66, 67]. Additionally, in sub-Sharan Africa over the past two decades, public health professionals and other health sector implementers in the healthcare sector have observed that inequity in wealth index remains a challenge in delivering healthcare services [68, 69].

The current study found mothers and caregivers from the younger age groups (15–24 years) were more likely to have zero-dose children in underserved settings of Ethiopia compared to mothers 25 years and older. This finding is consistent with prior studies conducted in similar contexts that showed younger mothers aged between 15 and 24 years and their current marital status could influence early child vaccination status [52, 70].

In addition, mothers who received less than four/no ANC and PNC follow-up care services were more likely to have zero-dose children, compared to mothers with four or more ANC visits. This echoes the findings from previous studies [70–72].

On the other hand, female-headed households as well as those who are employed mothers/caregivers are less likely to vaccinate their children [21, 73, 74].

Our results showed that children born to less- and medium-gender empowered women for decision making at the household level are over 1.63 and 1.71 times more likely to belong to the zero-dose category, respectively, compared to those born to women with a high level of empowerment/decision-making power in the social independence domain. Our findings are consistent with the existing literature and reported that in low- and middle-income countries, women’s empowerment improved child health and children of more empowered women are less likely to be left without vaccination [21]. In addition, more recently, Abreha et al. reviewed the evidence from sub-Saharan Africa and concluded that women’s empowerment, especially decision making and autonomy, was positively associated with child health outcomes [19, 75]. This suggests that aspects of empowerment related to autonomy and agency may be more relevant for achieving child immunization than maternal traits related to decision-making or attitude to violence [76].

This study has several strengths worth mentioning. Ascertainment of child immunization status was based on information gathered from multiple sources, which helped to verify and validate the results. We used a nationally representative and randomly selected sample from all the regions. This facilitated the generalization of results to all children in remote, underserved, or conflict-affected areas of Ethiopia. The use of digital applications and experienced data collectors helped to gather high-quality data. The results from this study could also be used for policy intervention in underserved settings in Ethiopia. It can also help push towards attaining the IA2030 and SDGs agenda.

This study also had limitations. The results from this study require cautious interpretation as it cannot be applied to all children in the general population. Noting the cross-sectional nature of the data, this study cannot establish causality. One limitation worth considering is the reliance on caregiver recall when immunization cards were not available. In such cases, the mother or caregiver was asked to self-report or recall the immunization status of the child. This method was used for approximately 39.2% of the children in our study. Even though, efforts were made to ensure thorough and accurate recall by providing clear instructions to caregivers and offering assistance in remembering important dates or events. It is important to note that this approach carries the potential risk of bias, specifically recall or information bias, particularly relevant for older children, as their immunization history may span over a longer period of time. Caregivers may find it more challenging to accurately recall immunization events that occurred many years ago. In such cases, the reliance on recall increases the risk of distorted information and potential bias.

### Public health implications and cues to action

This study highlights the crucial public health implications associated with the prevalence of zero-dose children in underserved regions of Ethiopia, which significantly deviates from the estimates provided by the WHO/UNICEF report. This stark contrast in numbers underscores the urgent need for immediate action to enhance vaccination coverage in these vulnerable communities. These findings offer valuable cues to action, enabling policymakers and healthcare professionals to design and implement targeted interventions that can effectively address the underlying barriers hindering the vaccination rates. By leveraging the insights gained from this study, comprehensive strategies can be developed to ensure that all children receive the vaccines they need to prevent potentially life-threatening diseases. Such targeted interventions can focus on the socio-economic factors, cultural beliefs, accessibility issues, and educational campaigns tailored to the specific needs of these communities. Additionally, collaborations with local healthcare providers, community leaders, and relevant stakeholders can play a pivotal role in establishing sustainable immunization programs that reach the most marginalized populations. By prioritizing the provision of vaccines and allocating sufficient resources, governments and organizations can work towards decreasing the number of zero-dose children and improving public health outcomes. This study’s significant findings not only point towards existing challenges but also provide an opportunity to understand the dynamics at play and implement evidence-based solutions that can lead to tangible improvements in vaccination coverage and ultimately safeguard the health and well-being of children in underserved areas. It is crucial to respond to these cues to action and collectively strive toward achieving universal immunization, thereby ensuring that no child is left without the vital protection vaccines offer.

## Conclusion and recommendations

To conclude, results from this national survey showed that the prevalence of zero-dose children in underserved and special setting populations of Ethiopia is very high. Wealth index, women empowerment, different context, single marital status, adolescent, and young maternal age groups, fewer than four ANC follow-up visits, and not receiving/utilizing PNC services were important predictors of zero-dose children in the study settings.

Therefore, empowering women and male engagement is one of the major recommendations to reduce zero-dose children in the study settings, as well as organizing tailored immunization service delivery targeted to hard-to-reach and remote areas. Additionally, we recommend integrating maternal and child health services with immunization services in all study settings, advocating and organizing targeted integrated and need-based “catch-up” campaigns/enhanced outreach vaccination services in conflict-affected areas, and regularly reaching conflict- and climate-affected areas and IDP centres through integrated service provisions of food, nutrition, and health services. Similarly, extending the hours of immunization services to meet parents’ needs (e.g., evening/weekend sessions) and continuing advocacy and community engagement activities to increase awareness of zero-dose children and support a community-owned, sustainable solution will help address barriers to reaching zero-dose children.

## Data Availability

Data used for this research is available from the corresponding author upon reasonable request.

## Declarations

**Author’s contributions**: WA & DA: initiated the study concept further enriched by SG, GAB, FS, TW, LA, MZ, YL, and KB. To the rest of the tasks, like study design, data collection, oversight, data interpretation, and manuscript writing all authors contributed equally. All authors have read and approved the manuscript.
